# The Amazonian Red Side-Necked Turtle *Rhinemys rufipes* (Spix, 1824) (Testudines, Chelidae) Has a GSD Sex-Determining Mechanism with an Ancient XY Sex Microchromosome System

**DOI:** 10.3390/cells9092088

**Published:** 2020-09-12

**Authors:** Patrik F. Viana, Eliana Feldberg, Marcelo B. Cioffi, Vinicius Tadeu de Carvalho, Sabrina Menezes, Richard C. Vogt, Thomas Liehr, Tariq Ezaz

**Affiliations:** 1 Instituto Nacional de Pesquisas da Amazônia, Coordenação de Biodiversidade, Laboratory of Animal Genetics, Av. André Araújo 2936, Petrópolis, Manaus CEP 69067-375, Brazil; Patrik.biologia@gmail.com (P.F.V.); feldberg@inpa.gov.br (E.F.); 2 Departamento de Genética e Evolução, Universidade Federal de São Carlos, São Carlos SP 13565-090, Brazil; mbcioffi@ufscar.br; 3 Universidade Regional do Cariri, Programa de Pós-Graduação em Diversidade Biológica e Recursos Naturais, Campus do Pimenta, Rua Cel. Antônio Luiz, 1161, Crato CEP 63105-100, Brazil; anfibios.repteis@gmail.com; 4 Universidade Federal do Amazonas, Laboratório de Evolução e Genética Animal, Instituto de Ciências Biológicas, ICB II, Setor Sul, Av. Rodrigo Octávio Jordão Ramos, 6200, Manaus CEP 69080-900, Brazil; 5 Instituto Nacional de Pesquisas da Amazônia, Coordenação de Biodiversidade, Centro de Estudos de Quelônios da Amazônia, Av. André Araújo 2936, Petrópolis, Manaus CEP 69067-375, Brazil; sabrina.bio@outlook.com (S.M.); vogt@inpa.gov.br (R.C.V.); 6 Institute of Human Genetics, University Hospital Jena, 07747 Jena, Germany; 7 Institute for Applied Ecology, Faculty of Science and Technology, University of Canberra, Canberra, ACT 12 2616, Australia; Tariq.Ezaz@canberra.edu.au

**Keywords:** genetic sex determination, comparative genome hybridization, Chelidae, Neotropical region, karyotype

## Abstract

The Amazonian red side-necked turtle *Rhynemis rufipes* is an endemic Amazonian Chelidae species that occurs in small streams throughout Colombia and Brazil river basins. Little is known about various biological aspects of this species, including its sex determination strategies. Among chelids, the greatest karyotype diversity is found in the Neotropical species, with several 2*n* configurations, including cases of triploidy. Here, we investigate the karyotype of *Rhinemys rufipes* by applying combined conventional and molecular cytogenetic procedures. This allowed us to discover a genetic sex-determining mechanism that shares an ancestral micro XY sex chromosome system. This ancient micro XY system recruited distinct repeat motifs before it diverged from several South America and Australasian species. We propose that such a system dates back to the earliest lineages of the chelid species before the split of South America and Australasian lineages.

## 1. Introduction

The side-necked turtles of the family Chelidae comprise over 60 valid species currently restricted to the southern hemisphere and can be found throughout Australasian and South American regions [1,2,3] Chelids stand out as the most diverse group of Pleurodira regarding their karyotype configurations, i.e., difference in ploidy numbers, varying numbers of *Mac* (macrochromosomes) and *mic* (microchromosomes), as well as the presence of sex chromosomes involving *Mac* and *mic* [4,5,6]. Despite such variability, some level of karyotype conservation is also evident. For example, except for one species, *Peltocephalus dumerilianus,* which has 2*n* = 26 [7], the Amazonian podocnemidids analyzed so far have 2*n* = 28 chromosomes, predominantly *Mac* type [8]. Even in chelids, which are the most speciose among Pleurodires, an evolutionarily conserved trend in karyotypes is visible. All Australasian species possess 2*n* = 50 or 2*n* = 54 chromosomes, for instance [4,6,9,10]. However, karyotypes of the South American lineages remain a puzzle, with several diploid numbers being described even in closely related species, as reported in *Mesoclemmys* species, which exhibit 2*n* = 40, 42, 52, 58, and 60 chromosomes [4,7,11].

The Amazonian red side-necked turtle (*Rhinemys rufipes*) is an exclusively Amazonian species that has a nocturnal lifestyle and occurs in small streams of close canopy forests, extending from the tributaries of the Apaporís river in Colombia throughout the upper Negro and Amazon river basins in Brazil [12,13,14,15,16,17,18,19,20]. However, little is known about many aspects of this species; for instance, the nesting sites of *Rhinemys* have yet to be discovered. Likewise, its sex determination strategies are still unknown, which, like the vast majority of Chelidae species across Australasia and South America lineages, may also share a genetic sex-determination (GSD) mechanism [9,10,21,22]. The stability of sex ratios for hatchlings in some Neotropical Chelidae species suggests GSD as a sex-determining mechanism for South American lineages [21,22,23,24]. For *Rhinemys*, however, it is not known whether the GSD mechanism is present or whether this species has an environmental sex determination (ESD). *Rhinemys* has a breeding season directly related to seasonal rain and precipitation patterns of the Amazon rainforest, which vary widely over the extent of its distribution [14,15,17,20]. This species is likely to use subterranean formations and galleries for nesting [19] at different seasons along the year [14,17,19], probably reflecting the non-stability of environmental variables (e.g., luminosity, temperature, and humidity). Overall, this suggests that these environmental features are not primary factors in the determination of the sex proportion of hatchlings in this species. The diploid number (2*n*) for Chelidae species ranges from 40 to 64 chromosomes, with the presence of well-differentiated *Mac* and *mic* sex chromosomes in some species [5,7,9,10,25,26]. However, the greatest karyotype diversity is found in the Neotropical species, with several 2*n* configurations, including cases of triploidy in the Neotropical twist-neck turtle (*Platemys platycephala,* 3*n* = 96) [4,5,7,25,26,27]. To date, only a single and contradictory reference exists concerning chromosomal data for *R. rufipes*: McBee et al. [5] reported data of 2*n* = 58 chromosomes and fundamental number NF = 64. However, this same study focused on the karyotypic composition of the *Acanthochelys* species, and no metaphase of *Rhinemys* was presented. Moreover, details regarding origin, sex, and number of individuals analyzed were not mentioned either. Therefore, reinvestigation of fresh material, with known gender and known origin individuals of *R. rufipes*, is required to corroborate these previous findings.

In this study, we aim to provide a full description of the karyotype composition of *R. rufipes* from the Amazon rainforest. For that, we applied multiple conventional (Giemsa staining and C-banding) and molecular cytogenetic tools, as comparative genomic hybridization (CGH), FISH mapping of telomeric (TTAAGG)*_n_*, 18S rDNA, and simple short repeats (SSRs) sequences. Additionally, we identified sex chromosomes and modes of sex determination in this species, and are thus able to discuss the evolution of sex chromosomes in the broader context of turtle sex chromosomes.

## 2. Material and Methods

### 2.1. Sampling, Mitotic Chromosomes Preparation, and C-Banding

Turtles were sampled from natural populations from the central Amazon region (Lower Rio Negro) under permission granted by Instituto Chico Mendes de Conservação da Biodiversidade (ICMBio) number 45275. We analyzed 5 males and 4 females of the red side-necked turtle *R. rufipes*. Chromosomal preparations were cultured from small blood samples at 29 °C, following Viana et al. [7]. C-positive heterochromatin staining was performed according to Sumner et al. [28]. It is highlighted that no animal needed to be euthanized in this study.

### 2.2. Probes for Chromosome Hybridization

18S rDNA and telomeric (TTAGGG)*_n_* probes were isolated according to Gross et al. [29] and Ijdo et al. [30], respectively. Both probes were labeled with aminoallyl-dUTPATTO-550 (red) by Nick-translation means (Jena Bioscience, Jena, Germany). Simple short repeats (SSRs), (GT)_15_, (AG)_15_, (AAC)_10_, (GGAT)_8_, (ACGC)_8_, (AATC)_8_, (ATCC)_8_, (AATG)_8_, (GACA)_8_, and (GATA)_8_ were used, directly labeled with *Cy-3*, during the synthesis [31].

### 2.3. Fluorescence In Situ Hybridization (FISH) for Repetitive DNA Mapping

FISH experiments followed the protocol described by Pinkel et al. [32], with modifications detailed in Viana et al. [33]. Briefly, the chromosome slides were denatured in 70% formamide/2xSSC at 70 °C; spreads were dehydrated in ethanol (100%). Then, 20 µL of the hybridization mixture (100 ng of each probe, 50% deionized formamide, and 10% dextran sulfate) was dropped on the slides, and the hybridization was carried out for 24 h at 37 °C in a moist chamber containing distilled water. The posthybridization washes were performed once in 2× SSC (44 °C, 5 min) and a final wash in 4× SSC/0.1% Tween (5 min, room temperature). The chromosomes were counterstained with DAPI (4′,6-Diamidino-2-Phenylindole, 1.2 µg/mL) and mounted in antifade solution (Vector, Burlingame, CA, USA).

### 2.4. Preparation of Probes for Comparative Genomic Hybridization (CGH)

The gDNA of males and females of *R. rufipes* was extracted from blood using the Wizard Genomic Purification Kit (Promega), following the manufacturer’s recommendations. Female-derived gDNA was labeled with aminoallyl-dUTPATTO-550 (red) and males’ gDNA with aminoallyl-dUTPATTO-488 (green) using a Nick-translation Labeling Kit (Jena Bioscience, Jena, Germany). The final hybridization mixture for each slide (20 μL) was composed of male- and female-derived gDNA (500 ng each), 20 μg of male-derived Cot-1 DNA (i.e., the fraction of genomic DNA enriched for highly repetitive sequences), prepared according to Zwick et al. [34], and the hybridization buffer containing 50% formamide, 2× SSC, 10% SDS, 10% dextran sulfate, and Denhardt’s buffer, pH 7.0. The probes were ethanol-precipitated, and the dried pellets were resuspended in hybridization buffer, as mentioned above.

### 2.5. Comparative Genomic Hybridization (CGH)

CGH experiments were performed according to our previous studies [35,36,37,38]. The slides were incubated at 37 °C in a dark, humid chamber for 72 h. Posthybridization washes were performed once in 50% formamide/2× SSC, pH 7.0 (44 °C, 5 min), once in 2× SSC (44 °C, 5 min), and a final wash in 4× SSC/0.1% Tween (3 min, room temperature). The chromosomes were counterstained with DAPI (1.2 µg/mL) and mounted in an antifade solution (Vector, Burlingame, CA, USA).

### 2.6. Microscopic Analyses

For the 5 males and 4 females, at least 10 metaphase spreads were analyzed for each in order to confirm the karyotype structure and FISH results. Images were captured using an Olympus BX51 microscope (Olympus Corporation, Ishikawa, Japan) with CoolSNAP. Chromosomes were classified as macrochromosomes (*Mac*) and microchromosomes (*mic*) or as metacentric (m), submetacentric (sm), subtelocentric (st), and acrocentric (a), according to [39].

## 3. Results

### 3.1. Karyotype and C-Positive Heterochromatin

**Figure 1:**
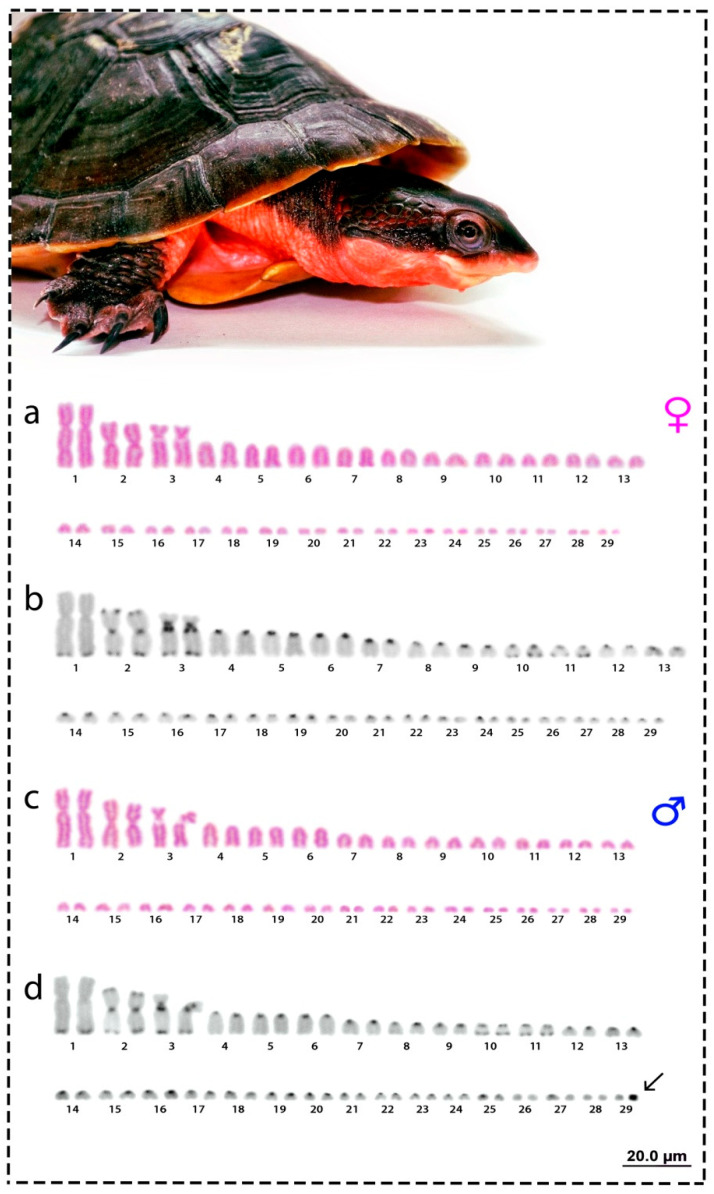
Karyotypes of females (**a**,**b**) and males (**c**,**d**) of *R. rufipes* in Giemsa-staining (**a**,**c**) and C-banding (**b**,**d**). Arrow indicates the tiny heterochromatic microchromosome, which corresponds to the Y sex chromosome. Bar = 20 µm.

The red side-necked turtle has 2*n* = 58 chromosomes, with 26 *Mac* and 32 *mic*. The karyotype is composed of 2m + 2sm + 2st + 20a and 32 *mic* (*mic* predominantly acrocentrics), with a fundamental number (NF) equal to 64 for males and females (Figure 1). The C-positive heterochromatin was found in all chromosomes, with a preferential localization in the centromeric regions (pairs 2 to 29) and in telomeric regions for some *Mac* pairs (1, 2, 3, 10, 11) in both males and females (Figure 1). However, one of the smallest *mic* in all males was completely heterochromatic and was not observed in any females. This identifies *R. rufipes* as having a XX/XY sex chromosome system (Figure 1).

### 3.2. Comparative Genomic Hybridization (CGH)

**Figure 2:**
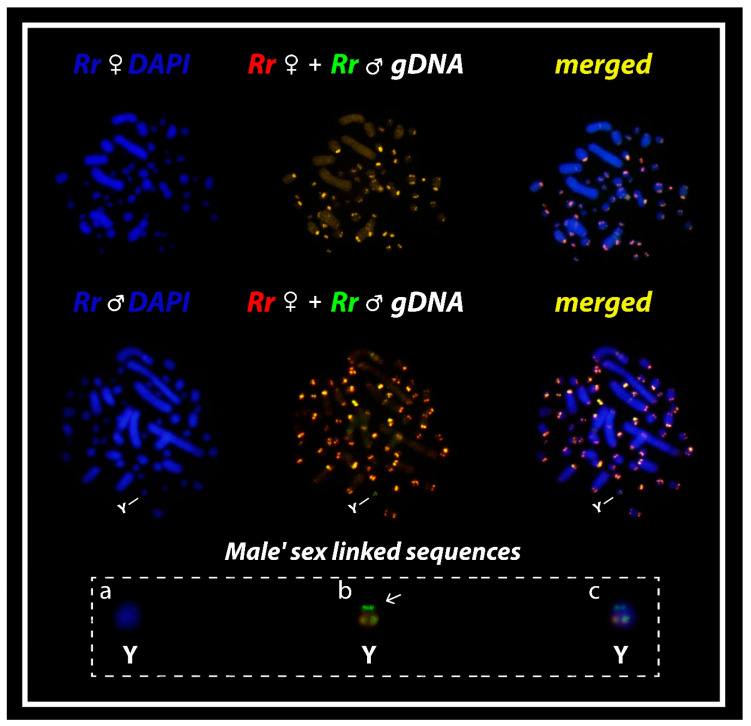
Mitotic chromosome spreads of *R. rufipes* after comparative genomic hybridization (CGH) procedures using male- and female-derived genomic probes. The common genomic regions are highlighted in yellow. The Y chromosome after DAPI (**a**), probed with male- and female-derived genomic probes (**b**) and merged image (hybridization signals + DAPI) (**c**), is highlighted in boxes. Please note the male-specific Y-linked sequences (**b**) in green.

Our intraspecific comparison between males and females of *R. rufipes* produced intense hybridization signals in both *Mac* and *mic*, mostly collocated with heterochromatic portions (Figure 2). The merged images identified male-specific sequences accumulated in a small region of a tiny *mic* of the karyotype, the micro Y sex chromosome (boxed). This identifies *R. rufipes* as a GSD species with a micro XY sex chromosome system. However, we are not able to properly identify the X chromosome due to the morphological similarity with many other *mics* and the absence of chromosomal-specific signal patterns (Figure 2).

### 3.3. Mapping of 18S rDNA, Telomeric Repeats and SSRs Motifs

**Figure 3:**
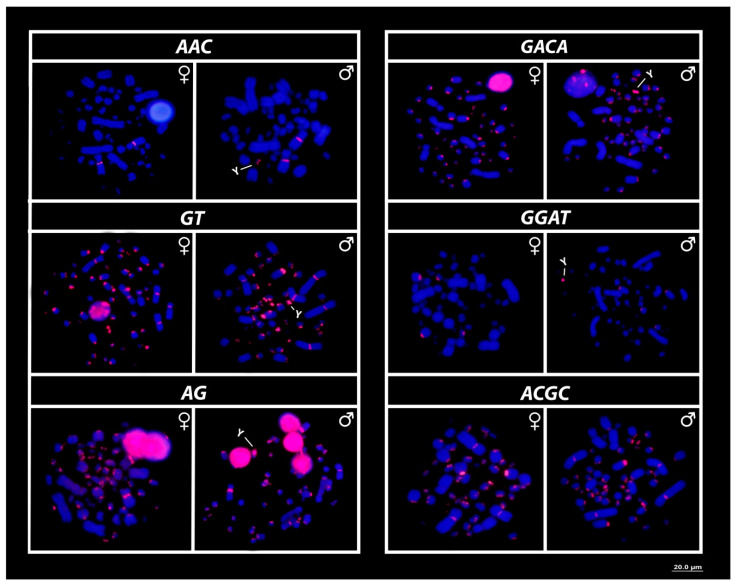
Mapping of several simple short repeats (SSRs) on the chromosomes of males and females of *R. rufipes*, with highlights to the particular accumulation for some of them on the micro Y sex chromosome (arrows). Bar = 20 µm.

**Figure 4:**
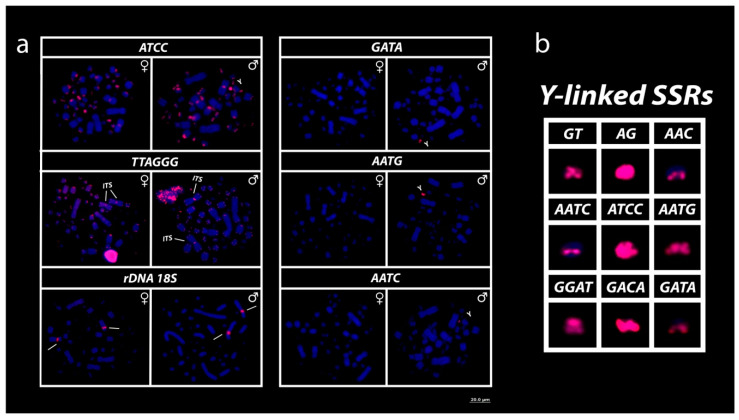
Mapping of several SSRs, telomeric (TTAGGG)*_n_*, and 18S rDNA on the chromosomes of males and females of *R. rufipes* (**a**). (**b**) Different patterns of SSR motif recruitment to enlarged forms of micro Y sex chromosome. Bar = 20 µm.

All microsatellite repeat motifs used, namely, (GT)_15_, (AG)_15_, (AAC)_10_, (GGAT)_8_, (ACGC)_8_, (AATC)_8_, (ATCC)_8_, (AATG)_8_, (GACA)_8_ and (GATA)_8_, showed hybridization signals on chromosomes of *R. rufipes* (Figure 3 and Figure 4). However, a Y-specific pattern of accumulation for some SSRs was observed (Figure 4b). The (GT)_15_, (AG)_15_, (ATCC)_8_, (GACA)_8_ and (GGAT)_8_ motifs showed hybridization signals on both *Mac* and *mic* of males and females, but with a particular amplification of such SSRs on the Y sex chromosome, nevertheless (Figure 3 and Figure 4). On the other hand, the motifs (AATC)_8_, (AATG)_8_ and (GATA)_8_ were found to be male sex-specific, with exclusive Y-linked amplification (Figure 4a,b). On the contrary, of all other SSRs used here, (ACGC)_8_ was the sole repeat motif with no differential pattern between males and females (Figure 3).

The mapping of the 18S rDNA sequences showed simple markings on the secondary constriction of the submetacentric pair (Figure 4). The telomeric motifs were evidenced on all terminal portions of all *Mac* and *mic*; however, an interstitial telomeric sequence (ITS) is evident on the centromeric region of the sole metacentric pair of the karyotype (pair 2; Figure 4).

## 4. Discussion

Although reptiles have been extensively studied from the cytogenetic point of view, the lack of karyotype data for many turtle species impairs a comprehensive analysis of evolutionary trends and their chromosomal relationships. In this sense, this study is the first to provide classical and molecular cytogenetic data for one representative of Chelidae species, the Amazonian red side-necked turtle *R. rufipes*. Our study demonstrates that this species has GSD with an XY sex microchromosome system, where the *mic* Y sex chromosome can easily be identified by the accumulation of SSRs. Moreover, our CGH experiments identified the presence of male-specific sequences concentrated on a small portion of the Y sex chromosome.

**Figure 5:**
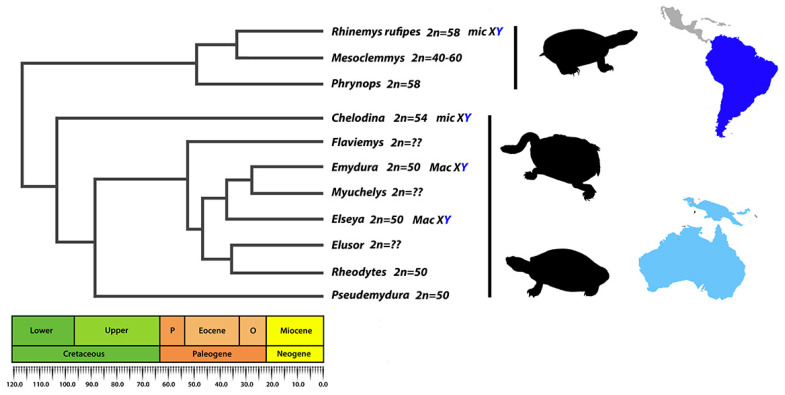
Chronogram for some chelid species, adapted from Pereira et al. [42]. P = Paleocene and O = Oligocene.

The side-necked turtles of the Chelidae family are phylogenetically related to Pelomedusidae (Africa) and Podocnemididae (Amazonia and Madagascar) turtle lineages, forming a monophyletic group (Pleurodira), predominantly widespread in the southern hemisphere [2,40,41,42,43]. Most Podocnemididae species (7 out of 8) exhibit 28 *Mac*, whereas Pelomedusidae species (for those with known karyotypes) present 34 and 36 chromosomes with the presence of *Mac* and *mic* [4,44,45,46]. Interestingly, the sole existing reference in the literature regarding the karyotype for *R. rufipes* [5] also reports 2*n* = 58 and NF = 64, very similar to our findings (Figure 1) [5]. This same karyotype configuration is also observed in some *Mesoclemmys* and *Phrynops* species [4,47], and although *R. rufipes* is an eccentric species among Neotropical chelids and easily recognized, given the paucity of additional information (e.g., locality of original samples or even the source of the samples), we cannot rule out the possibility that McBee and colleagues [5] have analyzed a *Mesoclemmys* or even *Phrynops* species, the latter inclusive, a genus in which *Rhinemys* was first placed. Nonetheless, *Phrynops* are older lineages than *R. rufipes*, whose origins date back ~34 mya, at the end of Oligocene (Figure 5), and much closer to *M. tuberculata*, *M. perplexa*, and *M. vanderhaegei*. However, despite presenting a GSD system [24,48], the configuration of sex chromosomes in the *Mesoclemmys* species needs further investigation to infer whether *Mac* or *mic* sex chromosomes frequently occur and whether transitions between *Mac* and *mic* are governing the karyotype diversity present in this group.

Sex chromosomes in turtles evolved independently and multiple times along its evolution [49], and several intra/interchromosomal rearrangements, sometimes involving a candidate for master sex-determining genes, are likely to represent the main factor that drove the landscape of sex chromosomes in turtles [50,51,52]. In Australasian chelids, for instance, translocations/fusion events orchestrated the origin of *Mac* and *mic* sex chromosomes in the ancestor of the species, with 2*n* = 50 and 2*n* = 54 [6,9,10,51,53].

The ancestor state for sex chromosomes in Australasia Chelidae is still under debate, namely, whether a *Mac* or *mic* ancestor sex chromosome was present before the split (~105mya) between the clade composed of species 2*n* = 50/*Mac* sex chromosomes and the clade with 2*n* = 54 and *mic* sex chromosomes (*Chelodina* spp.) (Figure 5) [4,6,9]. A *mic* sex chromosome ancestor state likely represents the most realistic scenario, which fused with a macro, resulting in the species with 2*n* = 50 and *Mac* sex chromosomes [53]. However, the reverse scenario is also plausible, suggesting that *Mac* ancestor could equally represent the ancestor condition, assuming *Chelodina* as the sister group to *Emydura* [6].

Australasian chelids comprise two major lineages, basically, a lineage with 2*n* = 50 and a lineage with 2*n* = 54. The *Emydura* species is closer to *Myuchelys* and *Elseya*, which forms a sister group to the lineages composed of *Elusor* and *Rheodytes*, jointly with *Flaviemys* and *Pseudoemydura*, species with *Mac* sex chromosomes and 2*n* = 50. All species with *Mac* sex chromosomes and 2*n* = 50 comprise a sister group to the *Chelodina* species that possesses 2*n* = 54 and *mic* sex chromosomes, representing the oldest lineages of Australasian chelids (Figure 5).

If *Elusor*, *Flaviemys*, and, especially, *Myuchelis* (sister group to *Emydura* spp. 2*n* = 50 and *Mac* sex chromosomes) somehow presented *mic* sex chromosomes, this scenario would require the occurrence of one turnover considering a *Mac* as the ancestral state and two turnovers considering a *mic* as an ancestor. Further analyses in *Myuchelys*, *Elusor*, and *Flaviemys* will uncover if these species also possess 2*n* = 50 and *Mac* sex chromosomes, as seen in their sister groups with known karyotype, and clarify the turnovers involving *Mac* and *mic* sex chromosomes in Australasian lineages.

Chelids, undoubtedly, possess South American origins, with fossil records dating to lower Cretaceous [2,54,55]; several studies also show that Chelidae (both South America and Australasia lineages) started to diversify before the split of Australasia from Gondwana [42,56,57,58]. This implies that besides vicariant events, they also experienced dispersal events, resulting in a widespread distribution at the beginning of the Gondwana fragmentation [2].

The *mic* sex chromosomes of *Rhinemys* date back to the early Oligocene, before the diversification of many Australasian species [42]. This suggests the possibility that *mic* sex chromosomes could already be present in the common ancestor of Australasian and South American chelids. In this scenario, an interesting question emerges as a new piece of the puzzle. If the *mic* sex chromosomes of *R. rufipes* somehow share homologies with the Australasia chelid sex chromosomes (*Mac* in the 2*n* = 50 species or *mic* in 2*n* = 54), this *mic* sex chromosome highlights ancestry dating back the Upper Jurassic, ~160 myr. This implies a much older turnover event involving *mic* and *Mac* sex chromosomes than the turnovers that occurred before the split of *Chelodina* and *Emydura* lineages [6,53]. Indeed, translocations involving a sex-determining locus via *mic* to *Mac* seems to have been the evolutionary pathway chosen by the chelids. However, further studies in other South American chelids are necessary and indispensable to investigate this hypothesis and decipher the real ancestor state for the sex chromosomes of chelids, as well as likely transition events involving macro and micro sex chromosomes along their evolutionary trajectories.

As seen in other chelids with poorly or highly differentiated sex chromosomes [6,53,59], *Rhinemys rufipes* also recruited some specific Y-linked SSR motifs (Figure 4b). These SSRs are commonly found on sex chromosomes across a range of Squamata reptile species [35,59] and are inherently thought to display a key role in chromosome sex differentiation [60,61,62,63]. Indeed, Squamata reptiles exhibit a dynamic genome regarding the rich content of SSRs [64,65,66,67]; however, turtles are relatively SSR-poor [67,68,69] even though sharing the same repeat motifs present in sex chromosomes of several Squamata lineages [59].

Regardless, *Rhinemys rufipes* and Australasia chelids share several SSRs on their sex chromosomes [6,59] despite a long divergence time, dating back to the split of South America and Australasia lineages in the Late Jurassic [42,70]. This could indeed represent a remnant of a common evolutionary history and origin of their sex chromosome. Chromosomics [71,72] is required to deeply infer the homology of sex chromosomes spanning ~115 myr of independent evolution among Chelidae species, especially because SSRs are polymorphic, showing high variability and elevated rates of mutation reflected in divergent microsatellites landscapes and rapid shifts even in closely related species [67]. In this sense, the presence of the recurrent recruitment of the same simple short repeats on the sex chromosomes of many reptiles, including *Rhinemys* and other chelid turtles, could simply be the product of co-optation and convergent accumulation of such repeats due to evolutionary advantages facilitated by the dynamic behavior of SSRs (e.g., recombination, control of genic expression, chromosomal organization).

SSR landscapes and their genome-wide distribution may inherently impact on chromosomal architecture; for instance, they are one of the major promoters of the expansion and/or contraction of repetitive sequences with which they are associated [73,74], for instance, transposable elements and rDNAs [63,75,76]. Here, we found at least three SSRs (GT, AG, AAC) bearing 18S rDNA of the 3rd pair, with minute differences in size, sometimes in both males and females (not shown in the figures), suggesting that these small repeats (di- and trinucleotides) are likely involved in regulatory processes, reflective of uneven crossing and ectopic recombination mediated by the association of such SSRs and the activity of the ribosomal sites. Interestingly, the other SSRs (the tetranucleotides: AATC, ACGC, GGAT, GACA), similarly present on both autosomes and sex chromosomes, did not present any association with the 18S rDNA sites, but had prominent signals on some small acrocentric chromosomes, perhaps displaying structural roles in the chromosomal architecture since they are mostly colocated with heterochromatic segments in both males and females (Figure 3 and Figure 4a).

Likewise, interstitial telomeric sequences (ITSs) can be found to be associated with rDNAs, several times attributed to represent hotspots prone to chromosomal rearrangements [77,78]. However, they are relatively rare in some turtle lineages [79]. Except for the Y-linked accumulation of telomeric motifs in *Elseya novaeguinae* [6], hitherto, TTAGGGn repeats on the centromeric position of the sole metacentric chromosome pair of *R. rufipes* (Figure 4) seem to be the first evidence of ITSs in Chelidae species. On the contrary, such repeats, as the remnant of chromosomal fusion events, is a widespread feature in its sister group, the Podocnemididae family [8]. In many vertebrate groups, ITS repeat has constantly been attributed to relics of ancient chromosomal rearrangements [33,80,81]; however, ITSs exhibit a random amplification across lineages [80,82], and, due to their dynamic functioning [78], sometimes do not represent artifacts or ghosts of past intra/interchromosomal rearrangements. Since the most of closely related species to *R. rufipes* also exhibits the 2*n* = 58, with very similar karyotype structure [5,7,42,47], the ITS present on the centromeric position of the sole metacentric pair could indeed be the result of past intrachromosomal rearrangements, but combined with the fact that these interstitial telomeric motifs are rarely found in some turtle lineages [79] and taking into account their dynamic behavior [78], it is also plausible that such ITSs may have arisen from other mechanisms, such as telomerase activity adding these motifs to nonterminal regions, duplication events, or even association with mobile elements and/or other repetitive sequences [83,84,85,86,87,88]. The karyotype of other South American chelids is also required to infer whether this ITS present in *R. rufipes* is a classical exception to the rule [82] or ITS sites are frequent and are shaping the karyotype diversity evident in Neotropical chelids.

The *mic* Y sex chromosome of *R. rufipes* can be easily identified by the accumulation of at least nine SSRs (Figure 4b); however, as with other chelid species possessing *mic* sex chromosomes, it was not possible to identify the *mic* X [6,59]. Undifferentiated sex chromosomes are notably frequent across the vertebrate phylogeny and are more common than previously thought [89,90,91]. Although the levels of degeneration are not primarily related to the age of the sex chromosomes and the lineages where they arose [92,93,94,95], they are more frequent in ancient lineages that did not acquire sex-specific signatures [37,96,97]. Interestingly, as revealed here in our study, even with ancient origin, they can also be found practically intact in recent lineages, where the X and Y of *R. rufipes* are undistinguished from each other, with the Y sex chromosome being identified solely by comparative genomic hybridization and recruitment of several SSR motifs. The sex chromosomes may remain undifferentiated for many reasons, for example, transitions and turnovers involving XY and ZW, evolutionary advantages promoted by the recombination between XY and/or Z and W, as well as shifts involving autosomes [37,38,39,40,41,42,43,44,45,46,47,48,49,50,51,52,53,54,55,56,57,58,59,60,61,62,63,64,65,66,67,68,69,70,71,72,73,74,75,76,77,78,79,80,81,82,83,84,85,86,87,88,89,90,91,92,93,94]. In this pathway, the biased male recruitment of repetitive sequences for the Y *mic* sex chromosome and the maintenance of the chromosomal morphology and similarity of the X and Y in *R. rufipes* seem to be another classical scape from the evolutionary trap of sex chromosomes [92,93,98].

The karyotype of *R. rufipes* surely brings important novelties to the knowledge of chromosome evolution and the evolutionary history of South America and Australasia Chelidae species. The likely ancestry for sex chromosomes also highlights that recruitment of particular SSR motifs on sex chromosomes could already have shaped the pathways that have resulted in the current configurations of *Mac* and *mic* sex chromosomes in chelids, long prior its diversification. Future analyses in other South America and Australasia chelids will uncover the events of rearrangements that the sex chromosomes underwent and decipher what scenario corresponds to the oldest sex chromosome configuration in Chelidae, namely, whether a *Mac* or a *mic* represents the ancestral state.
